# A Comparative Study of Educational Texts for Native, Foreign, and Bilingual Young Speakers of Russian: Are Simplified Texts Equally Simple?

**DOI:** 10.3389/fpsyg.2021.703690

**Published:** 2021-10-26

**Authors:** Anna Dmitrieva, Antonina Laposhina, Maria Lebedeva

**Affiliations:** ^1^Language and Cognition Laboratory, Pushkin State Russian Language Institute, Moscow, Russia; ^2^Faculty of Arts, University of Helsinki, Helsinki, Finland

**Keywords:** simple language, simple Russian, young readers, simplification strategies, textbook analysis, textbook corpus, text simplification, Russian language

## Abstract

Studies on simple language and simplification are often based on datasets of texts, either for children or learners of a second language. In both cases, these texts represent an example of simple language, but simplification likely involves different strategies. As such, this data may not be entirely homogeneous in terms of text simplicity. This study investigates linguistic properties and specific simplification strategies used in Russian texts for primary school children with different language backgrounds and levels of language proficiency. To explore the structure and variability of simple texts for young readers of different age groups, we have trained models for multiclass and binary classification. The models were based on quantitative features of texts. Subsequently, we evaluated the simplification strategies applied to readers of the same age with different linguistic backgrounds. This study is particularly relevant for the Russian language material, where the concept of easy and plain language has not been sufficiently investigated. The study revealed that the three types of texts cannot easily be distinguished from each other by judging the performance of multiclass models based on various quantitative features. Therefore, it can be said that texts of all types exhibit a similar level of accessibility to young readers. In contrast, binary classification tasks demonstrated better results, especially in the R-native vs. non R-native track (with 0.78 F1-score), these results may indicate that the strategies used for adapting or creating texts for each type of audience are different.

## Introduction

Modern data-based research on simple language and simplification is in critical need of sufficiently representative and reliable data—that is, texts that are samples of simple language. For the Russian language, this need is particularly acute. On the one hand, the concept of simple, easy, and plain Russian language has not been sufficiently investigated and is in its formative stages (Mustajoki et al., 2021). On the other hand, research on textual complexity in Russian is still in search of parameters that predict the complexity of comprehension more reliably and precisely than readability formulas (Laposhina, 2017; Solovyev et al., 2018). Psychophysiological studies of reading in the elementary school age confirm the influence of various factors, such as the frequency of words included in the text or discourse parameters of the text, on text comprehension, but at the moment these parameters are not considered in standard readability formulas for Russian (Petrova, 2016; Korneev et al., 2018, 2019). Finally, in the field of automatic simplification, a promising and actively pursued task in natural language processing, the problem of scarce data resources for simplification in Russian is noted (Sakhovskiy et al., 2021).

Any simple language research needs a relevant collection of samples of simple or simplified language. Such samples, on the one hand, are texts intended for developing readers. For example, Brouwers et al. (2014) employed educational materials such as encyclopedic entries simplified for children to study the strategies of sentence simplification in informative and narrative texts. In Gala et al. (2020), literary and scientific texts, along with their simplified versions, were used to create a parallel corpus for French learners who struggle with reading. During the creation of the Newsela corpus (Xu et al., 2015), the same texts were simplified for children at four different school grade levels to create a high-quality dataset for text simplification. All of these corpora can be used for the creation and/or evaluation of automatic text simplification systems. As for the Russian language, the linguistic complexity of texts for children was studied on educational materials for Russian-speaking students at primary school (Laposhina et al., 2019) and secondary school (Solovyev et al., 2018; Vakhrusheva et al., 2021) and the collection of book previews labelled with one of two categories—children's or adult (Glazkova et al., 2021).

On the other hand, most of the research on simplification is based on texts that were created or adapted for adult foreign language learners. According to Crossley et al. (2011), simplified L2 reading texts are either adapted from authentic texts or written explicitly for the L2 reader. The authors of textbooks are guided by educational standards and regulations, methodological experience and intuition, and non-formalized textual ideas that are simple enough to understand and affordable for non-native language learners. Such materials are used for studying the properties and text comprehension of simplified texts (Crossley et al., 2014) or in creating and testing simplification systems (Arfé et al., 2014). For the Russian language, texts for L2 learners were used for building systems of automatic complexity estimation (Karpov et al., 2014; Laposhina et al., 2018), refining objective parameters of text complexity (Solovyev et al., 2019), and studying L2 adaptation strategies (Sibirtseva and Karpov, 2014; Dmitrieva et al., 2021).

At the intersection of these two categories of simple texts are educational texts created for young L2 learners. Such texts constitute a separate category of simple texts, which are under-researched; usually, simplification studies are based either on texts for children or on texts for L2 learners.

Moreover, on the figurative scale of language proficiency, another category of children stands out—namely children with unbalanced bilingualism/multilingualism, including heritage speakers. In studies of Russian language acquisition and Russian language teaching practice, this category of children is identified specifically (Kagan and Dillon, 2003; Polinsky and Kagan, 2007; Protassova, 2008; Kalenkova and Zhiltsova, 2018; Moskovkin, 2019), and educational and assessment materials for such children are created and labelled separately from standard Russian young speakers, on the one hand, and from young L2 learners, on the other (Lebedeva et al., 2021). However, the specifics of texts written specifically for this category of children, and how they differ in complexity from texts for their peers with a different level of language proficiency, have not yet been studied.

Thus, the focus of our study is on three groups of texts for children with different language proficiency in Russian. A detailed study on the arrangement and simplicity of such data is of significant importance for studying simplification strategies, and it may contribute to both research of text complexity and the field of language teaching.

## Research Questions

The study aims to explore the simple Russian language presented in texts for children with different levels of language proficiency. Herein, we determine which simplification strategies are used to create simple texts for different groups of readers.

This study hypothesizes that the target group of simplification (children or second language learners) determines simplification strategies, so that simple texts for different groups of readers are modified differently.

In this study, we test the hypothesis on educational texts for children with the different settings of the Russian language acquisition such as follows: primary school children with Russian as a native language (hereafter R-native), their peers with Russian as a weaker language in unbalanced bilingualism (hereafter R-bilingual), and children who study Russian as a foreign language outside the Russian language environment (hereafter R-foreign).

Accordingly, this study aims to answer the following research question:

Are there any specific simplification strategies in educational texts for children with different language backgrounds and levels of Russian language proficiency?

## Materials and Methods

### Corpus Building

To answer the aforementioned research questions, we employed Text-Image Russian Textbook Corpus (TIRTEC) of texts from Russian language textbooks for children aged 7–11 years (corresponding to the age of primary school students in the Russian education system), intended for three groups of children based on their language proficiency and settings of language acquisition: R-native, R-bilingual, and R-foreign^1^. We followed the existing division of texts into the three target groups and relied on the methodological description of the target audience of the textbook indicated by the authors in the book annotation (e.g., “for bilingual 10-year-olds learners Russian at weekend schools”).

Table 1 shows the volume and basic text characteristics of the three groups of texts randomly chosen from the TIRTEC corpus for the following experiment. Each group contains the same number of texts, 1,100, so that the classes were balanced for future experiments. The texts for the R-bilingual group contain the maximum number of words and many unique words, whereas the least number of words is found in the R-native texts. This is due to the peculiarities of the Russian school system, in which the Reading course has separate textbooks that were not included in the TIRTEC corpus, while textbooks for R-bilingual and R-foreign combine linguistic exercises and reading in one book.

**Table 1:** Characteristics of the three subdomain of texts randomly chosen from the TIRTEC corpus for the following experiment.

	**R-foreign**	**R-bilingual**	**R-native**
**Collection size**			
Number of texts	1,100	1,100	1,100
Number of tokens	39,955	58,964	31,670
Vocabulary size (number of unique tokens)	8,846	12,760	10,919
**Text source**			
Simple fragment of authentic text	170	205	727
Fragment of authentic text adapted by textbook authors	61	41	30
Texts written specifically for this textbook	869	854	343
**Basic text characteristics**			
Mean sentence length (words)	5.84	7.3	7.56
Mean word length	4.67	4.86	5.14
Average number of punctuations per sentence	0.73	0.81	1.02

Each domain includes texts from the different sources: fragments of authentic text (e.g., written by Pushkin A.); fragments of authentic text adapted by textbook authors (e.g., based on “The Tale of the Fisherman and the Fish” by Pushkin A.); and texts written specifically for this textbook. However, the proportion of these types differs among these three groups, which can also be an illustration of different strategies for simple text selection.

In terms of language proficiency, these groups should form an ascending scale of language users, from beginners (R-foreign) to proficient (R-native), according to their age; R-bilingual children are expected to occupy a middle position. This is confirmed by the average word and sentence length, and the average number of punctuation symbols per sentence.

### Text Preprocessing

First, texts from coursebooks were digitized and annotated with meta-attributes manually. Before extracting text features for feature-based models, we cleaned the texts of noisy symbols and non-standard punctuation (for example, we replaced “?.” with “?”). Before extracting some features, such as coverage by different word lists, we also lemmatized the texts with the Mystem 3.1 toolkit for Python (Segalovich, 2003). Sentence tokenization was performed with ru_punkt^2^, an NLTK sentence tokenizer for Russian.

### Features Extraction

We identified a **set of quantitative features** that determine the difficulty level of the text, building on relevant research on automated readability assessment (Karpov et al., 2014; Reynolds, 2016; Laposhina et al., 2018, Sharoff et al., 2008). Our current study makes use of 95 features which can be divided into four groups.

1. ** Length-based features** of texts are presented by average word and sentence length and the ratio of words longer than four syllables.
2. ** Readability formulas**. We implement the 5 often used in modern Russian readability studies formulas:

- Flesch–Kincaid readability tests
- The Coleman–Liau index
- Dale–Chall readability formula
- Automated Readability Index(ARI)
- Simple Measure of Gobbledygook (SMOG)

Almost all of them represent various combinations of mean word length in signs or syllables, sentence length, and constant coefficients.

3. **Lexical features** include:

- Coverage by vocabulary lists for the learners Russian as a foreign language graded by the Common European Framework of Reference for Languages (CEFR) levels (Andryshina and Kozlova, 2012, 2015; Andryshina, 2017a,b). Since there are currently no such lists specifically for children, we used the version for adult learners of Russian
- Coverage by frequency lists of Modern Russian Frequency Dictionary (Lyashevskaya and Sharov, 2009)
- Coverage by the list of abstract words
- Type/token ratio (TTR) is the ratio of different unique word stems (types) to the total number of words (tokens) that indicate lexical diversity in the text
- Lexical density is calculated as the ratio of lexical items to the total number of words.

4. **Morphosyntactic features** represent the relative ratio of tokens with given morphosyntactic tags, so observed frequencies of POS tags were divided by the total amount of words in the text (e.g., the number of NOUN-tags divided by the total number of tokens), counts of cases were divided by the number of words that have cases. We used 50 morphosyntactic tags in total, e.g., percentage of nouns, prepositions, conjunctions, words in the genitive case, and the number of passive forms.

Features from groups 1, 3, 4 were extracted using Python programming language and the Mystem 3.1 toolkit. Readability formulas with constants optimized for Russian texts were taken from I. Begtin's Plain Russian project^3^.

### Model

To study the possible relations between various features of texts and their domains, we employed both multiclass and binary classification, using Python and the scikit-learn library (Pedregosa et al., 2012) to build our models^4^. Scikit-learn allows for simple and efficient data analysis with the help of many built-in tools such as various statistical models. For the multiclass setup, we used multinomial logistic regression with a limited-memory Broyden-Fletcher-Goldfarb-Shannon (BFGS) solver (“LBFGS”) for optimization. For binary tasks, we employed logistic regression with default parameters. We also scaled all features between 0 and 1 during preprocessing when working with our text metrics.

### Model Testing

To test the adequacy of the model and extracted features, we trained a binary model on two groups of texts with obvious differences in simplicity and comprehensibility: texts from our corpus for children of primary school age vs. fragments from fiction books included in the high school curriculum (such as War and Peace by L. Tolstoy, and Oblomov by Goncharov), with a similar total word count. Both models showed high performance in the classification tasks: model showed an F_1_-score of 90 (see Table 2). This demonstrates that the selected sets of features can distinguish texts by difficulty level, and also confirms the general presupposition that the texts we have selected actually are the examples of a simple language.

**Table 2:** F1-scores in binary feature based classifiers.

**Classifier**	**F1-score**
R-native vs. non-R-native	0.78
R-foreign vs. non-R-foreign	0.72
R-bilingual vs. non-R-bilingual	0.68
R-native adult vs. R-native kids	0.9

## Results

As can be seen in Table 1, one of the parameters by which the three groups differ is the source of the educational text. While simple fragments in R-native textbooks are mostly taken from children's and classical literature, many of the R-foreign and R-bilingual texts are written specifically for educational purposes. Authentic texts are most often presented in these books in small folklore genres: songs, riddles, and proverbs.

### Regression Models

To estimate the homogeneity of texts within the three selected groups in terms of their linguistic features, we performed both multiclass and binary classifications based on text features described in section Features Extraction.

**Figure 1:**
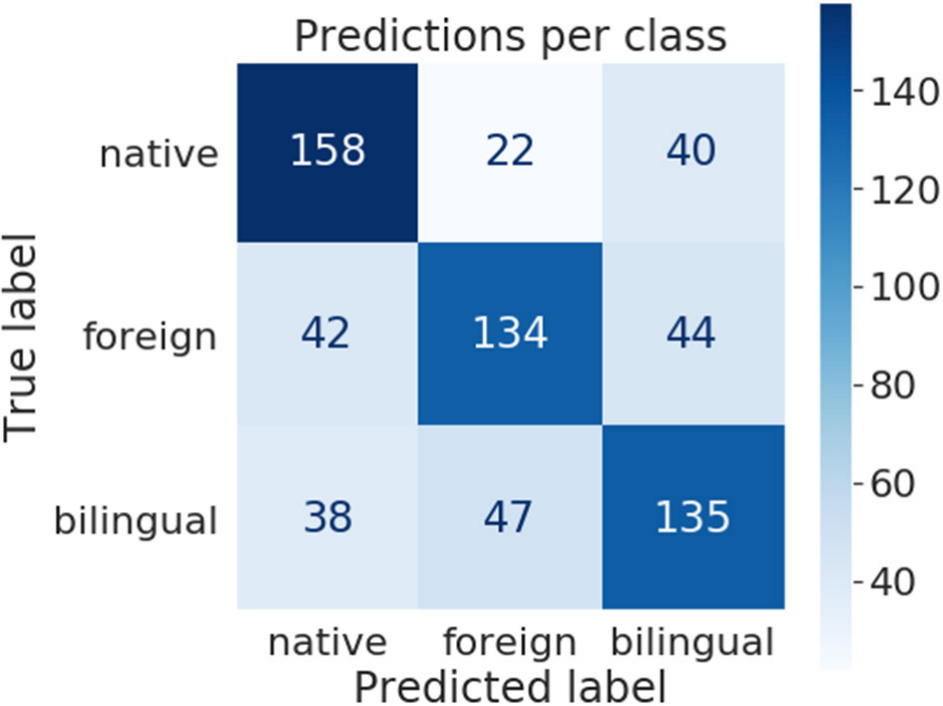
Confusion matrix of multiclass model predictions based on set of text features.

**The multiclass model** task was to predict a right target audience for the given text—R-native, R-foreign, or R-bilingual. It performed best on R-native texts with an F_1_-score of 69, and worst on R-bilingual, with an F_1_-score of 62, but these results are not satisfactory enough. As can be seen in Figure 1, most of the time, the model confused R-bilingual texts for R-foreign and *vice versa*. The classification task of predicting the target audience for educational text written for young learners of Russian proved to be difficult.

However, transforming the task into **several binary classifiers** improved accuracy. We performed a series of binary comparisons of these collections. In these one vs. the rest setups we tried to train the models to distinguish one particular class from the rest of the texts: for example, R-native texts from non-R-native (R-foreign and R-bilingual texts combined) texts. The numbers of instances in classes 1 and 0 were equal.

The best results were observed in the R-native vs. non-R-native comparison with a 78 F_1_-score for class 0 and 77 for class 1 (see Table 2). The ROC curves for one-vs-rest setups were again best in the R-native classifier with an AUC score of 0.85, and worst in the R-bilingual classifier with an AUC score of 0.73.

The model error analysis shows that for all types of errors, the median value of the percentage of words from lexical minima turns out to be closer to the median value not of its correct category, but of the one determined by the model. For instance, texts that were marked R-bilingual by the model while actually being R-native tend to contain more vocabulary from the CEFR-graded lexical minima than R-native texts contain on average. And in texts marked R-foreign instead of R-native these numbers were even higher. This can indicate that lexical differences were one of the factors that confused the model. Readability proved to be among such factors as well. Some grammatical features, such as relative numbers of adjectives, nouns, verbs and adverbs among all words, also influenced wrong decisions of the model. For example, in R-native texts the relative number of adjectives is quite high on average. However, in R-native texts that were wrongly identified as R-bilingual this number is lower, and in R-native texts marked as R-foreign there were almost no adjectives at all. Finally, it is worth noticing that the model made more errors on texts from certain textbooks, which may indicate that these texts do not correspond to the proclaimed target audience. It is especially true for the most diffuse category, R-bilingual.

### Correlations and Means

To analyze the effect of each text feature for the texts discrimination into three groups, we examined correlations on our data using Kendall's tau coefficient. This non-parametric test does not rely on assumptions about variable distributions. We assumed the text features to be independent variables, and the class of text (R-native, R-foreign, or R-bilingual) to be the dependent variable.

The results of hypothesis testing are shown in Table 3. We tested the correlations on binary problems; for example, the correlations of the features in the R-foreign section are calculated using a binary dependent variable, where 1 is R-foreign texts and 0 is non-R-foreign texts (the numbers of entries in each class are balanced).

**Table 3:** Selected Kendall's tau correlations between the dependent variable (class) and various independent variables (features).

**Domain**	**Most significant features**	**Kendall's τ**
R-foreign	Relative numbers of verbs in past tense	−0.28
	Percentage of A1 vocabulary	0.28
	Percentage of A2 vocabulary	0.26
	Coleman's readability formula	−0.25
	Percentage of B1 vocabulary	0.25
	Relative numbers of verbs in perfective aspect	−0.24
R-bilingual	Number of unique words	0.19
	Number of words	0.19
	Text coverage by 5,000 most frequent Russian words list	0.18
	Relative amount of nouns	−0.13
	Lexical density	−0.12
	TTR	−0.12
R-native	Percentage of A1 vocabulary	−0.39
	Percentage of B2 vocabulary	−0.39
	Percentage of B1 vocabulary	−0.38
	TTR	0.33
	Coleman's readability formula	0.26
	ARI readability formula	0.25

*P-value of all counts < 0.05*.

It should be admitted that we did not observe any particularly strong correlations here. However, we note some peculiarities that may be associated with different strategies for creating and adapting education materials for these groups of children. First, the coefficients among these groups are different: the highest coefficients are observed in the R-native group, and the lowest one—in the R-bilingual, which may indicate the heterogeneity of this group. The top lines of the table for groups R-foreign and R-native are occupied by features based on lexical minima for adult L2 Russian learners. It may be a signal of a difference in understanding of simple basic vocabulary among these groups. R-native textbooks contain texts from Russian classical literature, prose about nature as well as children literature—this leads to the presence in books of specific vocabulary about nature and agriculture (e.g., оляпка “white-throated dipper,” осина “aspen,” элеваmop “grain elevator”). At the same time, the text materials for the R-foreign group are more guided by designated lexical minima for L2 learners, which contain more everyday vocabulary. Meanwhile, in R-bilingual texts features based on lexical minima did not play a significant role. However, other lexical indicators came to the fore, such as words from frequency lists, lexical density, and lexical diversity.

A negative correlation between the number of verbs in the past tense and the R-foreign group (the more such verbs in the text, the less likely it is that the text belongs to the R-foreign group) may be due to the simplicity in grammatical forms: foreign students start using verbs from the present and future tense forms. The relative numbers of verbs in the perfective aspect, which do not have present tense forms in Russian, also speak in favor of this hypothesis. It can also be caused by the fact that textbooks for foreigners have a large number of examples of everyday communicative situations, in contrast to fiction texts for R-native, which is often turned to the past.

## Discussion and Conclusion

The study revealed that the three types of texts cannot easily be distinguished from each other by judging the performance of multiclass models. Therefore, it can be said that texts of all types exhibit a similar level of accessibility to young readers. In contrast, the feature-based approach proved to be effective at binary tasks, especially in the R-native vs. R-foreign tracks. These results indicated that the strategies used for adapting or creating texts for each type of audience are different, which makes some groups of texts easier to distinguish. For instance, in R-foreign texts, more standardized words are used, and conversely, in R-native texts the vocabulary is richer, and more advanced grammar is used. The considerable difference between the R-native domain and the others can also be explained by the number of authentic texts in this part of the corpus, as opposed to the R-foreign and R-bilingual domains, in which texts written specifically for textbooks are common. Judging by the correlation analysis, it seems that texts intended for R-foreign learners contain fewer verbs in past tense forms, which may indicate different notions about the grammatical side of the text complexity. The most informative lexical features for R-native and R-foreign groups were those based on lexical minima for adult L2 Russian learners. This suggests that authors of educational texts for foreign children are largely guided by the requirements of the CEFR level system, although these requirements have not been accommodated to children studying Russian. The text coverage by lexical minima of R-bilingual text is higher than the R-native group, even considering that, for example, R-bilingual texts are longer on average. The R-bilingual group showed a low connection with the linguistic parameters of the text in the binary classification task (R-bilingual vs. not R-bilingual). Therefore, we can assume that this group is the most diverse, combining different strategies and views on text simplification. The complexity and heterogeneity of this group of texts create significant limitations for the use of these materials as data for simplification outside the field of research on heritage speakers and bilinguals.

The experiments described above were limited to examining the differences between the three domains of the simplified Russian language. In future studies, it would be interesting to investigate the change in the comprehensibility level inside these domains, for example, from one school grade to another, and to observe whether the language of educational texts reflects a crucial restructuring in reading patterns that occurs around the third grade (Korneev et al., 2019).

Overall, the study found that the three observed domains can be ordered on a scale from the simplest (R-foreign with simpler grammar and standardized vocabulary) to the most complex (R-native with a richer vocabulary and more complicated grammar). Despite the fact that in the practice of Russian teaching educational materials for bilinguals are distinguished as a separate category, the quantitative linguistic analysis showed that the status of R-bilingual texts is ambiguous and they are the least classified area. The results of this linguistic study contribute to various areas of research on simple Russian and suggest directions for further research, including psychophysiological research aimed at studying which text parameters constitute complexity for different categories of young readers with different levels of Russian language proficiency.

## Data Availability Statement

Publicly available datasets were analyzed in this study. This data can be found here: https://digitalpushkin.tilda.ws/tirtec.

## Author Contributions

AD: literature review, conducting preprocessing, classification model building and evaluation, data analysis, and writing the paper. AL: data collection and annotation, data analysis, interpretation of results, and writing the paper. ML: conception and design of the study, formulation of research goals and aims, literature review, interpretation of results, writing the paper, overall management, and coordination of the study. All authors contributed to the article and approved the submitted version.

## Funding

This research has been supported by the RFBR Grant No.17-29-09156.

## Conflict of Interest

The authors declare that the research was conducted in the absence of any commercial or financial relationships that could be construed as a potential conflict of interest.

## Publisher's Note

All claims expressed in this article are solely those of the authors and do not necessarily represent those of their affiliated organizations, or those of the publisher, the editors and the reviewers. Any product that may be evaluated in this article, or claim that may be made by its manufacturer, is not guaranteed or endorsed by the publisher.

## Footnotes

^1^Textbook names and references are available at https://digitalpushkin.tilda.ws/tirtec.

^2^ https://github.com/Mottl/ru_punkt

^3^ https://github.com/infoculture/plainrussian

^4^The source code is available on https://github.com/Digital-Pushkin-Lab/SimplifiedRussianTextsForChildrenClassif.
